# Time-specific microRNA changes during spinal motoneuron degeneration in adult rats following unilateral brachial plexus root avulsion: ipsilateral vs. contralateral changes

**DOI:** 10.1186/1471-2202-15-92

**Published:** 2014-07-24

**Authors:** Ying Tang, Ze-Min Ling, Rao Fu, Ying-Qin Li, Xiao Cheng, Fa-Huan Song, Hao-Xuan Luo, Li-Hua Zhou

**Affiliations:** Department of Anatomy, Zhongshan School of Medicine, Sun Yat-sen University, No. 74 Zhongshan Road 2, Guangzhou, 510080 P.R. China; Department of Radiology, The Fifth Affiliated Hospital, Sun Yat-sen University, No. 74 Zhongshan Road 2, Guangzhou, 510080 P.R. China

**Keywords:** Brachial root avulsion, MicroRNA, Microarray, Inflammatory response, nNOS, c-jun, ATF-3, Calpain 2

## Abstract

**Background:**

Spinal root avulsion induces multiple pathophysiological events consisting of altered levels of specific genes and proteins related to inflammation, apoptosis, and oxidative stress, which collectively result in the death of the affected motoneurons. Recent studies have demonstrated that the gene changes involved in spinal cord injury can be regulated by microRNAs, which are a class of short non-coding RNA molecules that repress target mRNAs post-transcriptionally. With consideration for the time course of the avulsion-induced gene expression patterns within dying motoneurons, we employed microarray analysis to determine whether and how microRNAs are involved in the changes of gene expression induced by pathophysiological events in spinal cord motoneurons.

**Results:**

The expression of a total of 3,361 miRNAs in the spinal cord of adult rats was identified. Unilateral root-avulsion resulted in significant alterations in miRNA expression. In the ipsilateral half compared to the contralateral half of the spinal cord, on the 3rd day after the injury, 55 miRNAs were upregulated, and 24 were downregulated, and on the 14th day after the injury, 36 miRNAs were upregulated, and 23 were downregulated. The upregulation of miR-146b-5p and miR-31a-3p and the downregulation of miR-324-3p and miR-484 were observed. Eleven of the miRNAs, including miR-21-5p, demonstrated a sustained increase; however, only miR-466c-3p presented a sustained decrease 3 and 14 days after the injury. More interestingly, 4 of the miRNAs, including miR-18a, were upregulated on the 3rd day but were downregulated on the 14th day after injury.

Some of these miRNAs target inflammatory-response genes in the early stage of injury, and others target neurotransmitter transport genes in the intermediate stages of injury. The altered miRNA expression pattern suggests that the MAPK and calcium signaling pathways are consistently involved in the injury response.

**Conclusions:**

This analysis may facilitate the understanding of the time-specific altered expression of a large set of microRNAs in the spinal cord after brachial root avulsion.

**Electronic supplementary material:**

The online version of this article (doi:10.1186/1471-2202-15-92) contains supplementary material, which is available to authorized users.

## Background

Brachial root avulsion is a type of injury that leads to motor function loss as a result of motoneuron degeneration. Previous studies have shown that avulsion-induced motoneuron damage is propagated by a cascade of molecular and cellular events including changes in the expression of genes and the phosphorylation of signaling molecules in cell death-related pathways [1–3]. Based on the microarray analysis of the affected spinal cord after root avulsion in previous studies, the downregulation of genes required for promoting neuronal survival and axonal regeneration and the upregulation of genes involved in apoptosis and DNA damage were observed. Furthermore, our recent studies showed that some avulsion-induced genes, such as neuronal nitric oxide synthase (nNOS), showed changes at the mRNA level that were different from the changes at the protein levels [4, 5], whereas other changes, such as those in c-jun, were similar at the protein and mRNA levels [1, 3]. However, the upstream and downstream molecular mechanisms of avulsion-induced abnormal gene expression during motoneuron degeneration are still unclear and need to be studied further. MicroRNAs (miRNAs) are small non-coding RNAs that are key determinants of mRNA stability [6]. miRNAs modulate protein expression levels by antagonizing mRNA translation and are powerful regulators of cellular function [7]. Individual miRNAs target and block hundreds of protein-coding genes [8] that regulate many biological processes in neuronal lesions [9]. An increasing number of studies have demonstrated that miRNAs in the spinal cord are altered in a variety of motor neuron degenerative diseases and after spinal cord injury [8, 10–12]. There is also emerging evidence that alterations in RNA metabolism in the spinal cord are time-specific and may be critical in the progression of avulsion-induced motoneuron degeneration [3, 13]. However, little is known regarding the miRNA expression profile in the spinal cord during avulsion-induced motoneuron degeneration. Therefore, we hypothesize that the use of miRNAs may be an ideal and potent method for determining the underlying mechanism of avulsion-induced motoneuron death. In the present study, we investigated the miRNA expression patterns based on the time course of motoneuron death by using microarray analysis followed by quantitative RT-PCR confirmation. We chose two time points, 3 and 14 days after avulsion, when the RNA stability in the spinal cord was lost and when motoneuron death began to occur, respectively, to investigate the miRNA expression patterns. Furthermore, we focused on the changes in the miRNA expression patterns in the ipsilateral vs. contralateral halves of the affected spinal cord. We employed bioinformatics analysis to determine the functional roles of target genes regulated by altered miRNAs, which further suggested the effects of miRNA dysregulation on the key processes of the brachial root avulsion injury.

## Results

### MicroRNA expression profiling of the injured rat spinal cord

**Figure 1:**
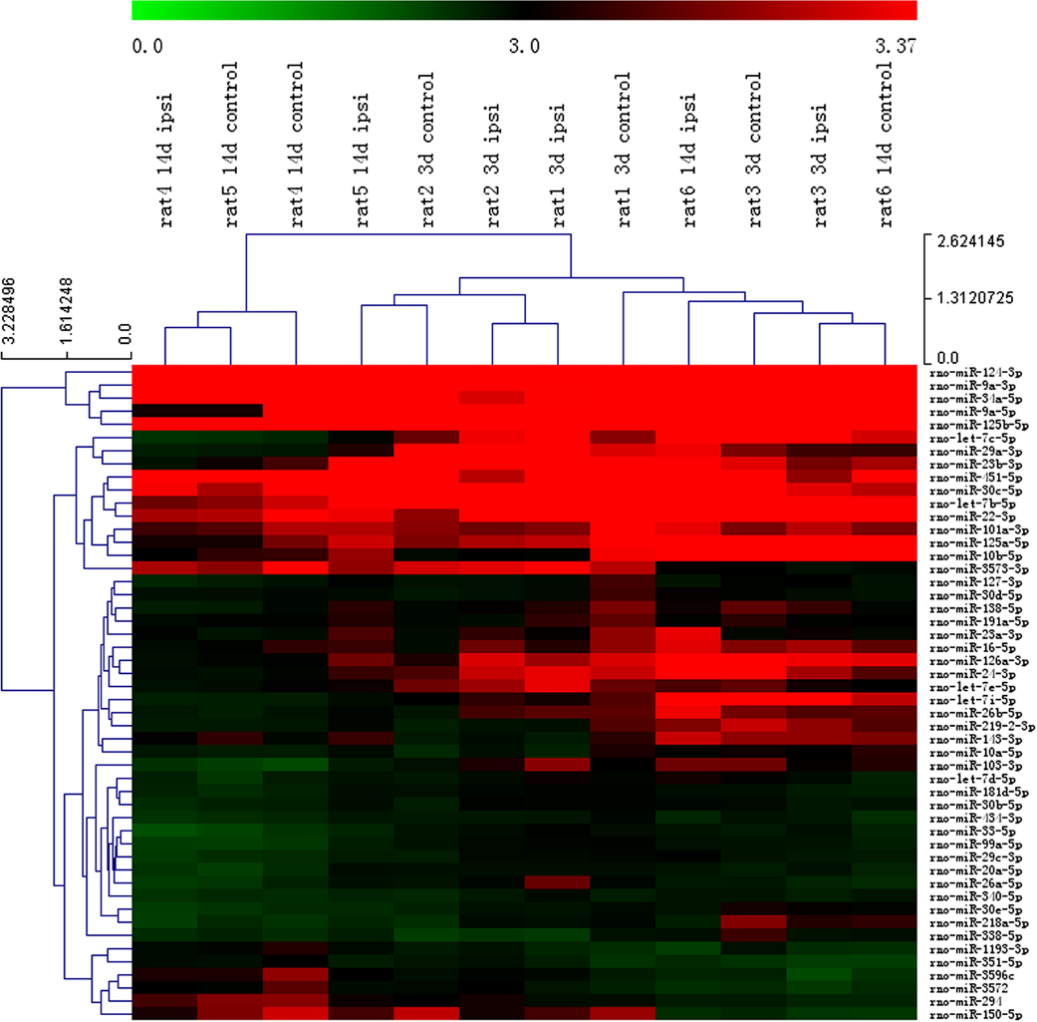
**MicroRNA expression profiles after ventral combined with dorsal root avulsion.** Heat map generated from a selection of the 50 most highly expressed miRNAs with levels of intensity >30 in each rat that underwent an avulsion injury and were sacrificed 3 days and 14 days after injury comparing the ipsilateral to the contralateral sides. Green color indicates a low relative expression level, and red indicates a high relative expression level.

**Figure 2:**
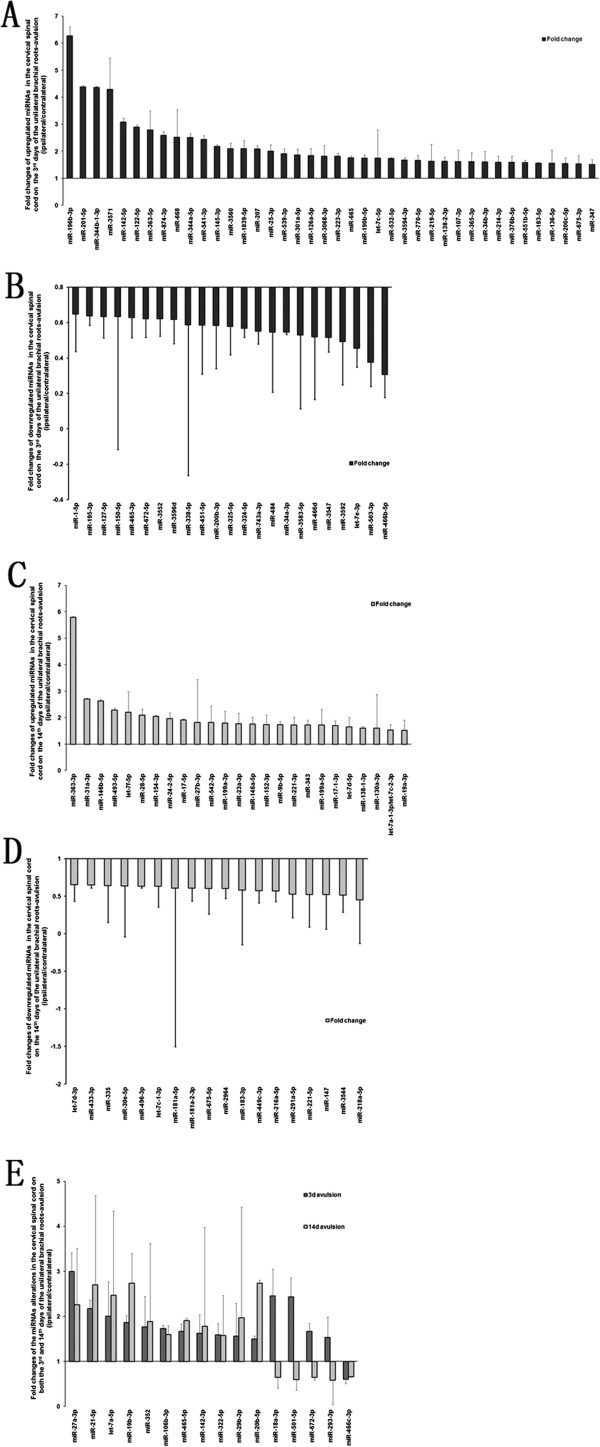
**Significant expression changes in the microRNAs on the 3rd day and 14th day after injury. (A)** miRNAs significantly upregulated on the 3rd day. **(B)** miRNAs significantly downregulated on the 3rd day. **(C)** miRNAs significantly upregulated on the 14th day and **(D)** significantly downregulated on the 14th day. **(E)** miRNAs that were consistently significantly changed on the 3rd day and the 14th day.

In the present study, 3,361 miRNAs were expressed in the cervical spinal cord of the adult rats. After normalizing the signal intensities for all miRNA expression levels, miR-124-3p, miR-9a-3p, miR-34a-5p, miR-9a-5p, miR-125b-5p, miR-let-7c-5p, miR-29a-3p, miR-23b-3p, miR-451-5p, and miR-30c-5p were the miRNAs expressed at the highest levels (Figure 1). In the rats with right brachial plexus root avulsion, the miRNA expression patterns of the ipsilateral spinal cord were significantly changed compared to those in the contralateral cervical spinal cord (Figure 2). Ventral combined with dorsal root avulsion resulted in a sustained upregulation of 10 miRNAs, including miR-19b-3p, miR-20b-5p, miR-21-5p, miR-27a-3p, miR-29b-3p, miR-106b-3p, miR-142-3p, miR-322-5p, miR-352, and let-7a-5p (Figure 2E). Four of the miRNAs, including miR-18a-3p, miR-293-3p, miR-501-5p, and miR-672-3p, were upregulated on the 3rd day but downregulated on the 14th day after the injury (Figure 2E). Only miR-466c-3p was continuously downregulated on both the 3rd and 14th days after the injury (Figure 2E). Furthermore, 40 of the miRNAs, including miR-34b-3p, miR-25-3p, miR-126-5p, miR-142-5p, and miR-324-5p, were only transiently upregulated (Figure 2A); the other 23 miRNAs, including miR-34a-3p and miR-324-5p, were transiently downregulated on the 3rd day but returned to normal levels by the 14th day (Figure 2B). On the 14th day after injury, 25 miRNAs, including miR-31a-3p, miR-17-5p, miR-146b-5p, miR-154-3p, and miR-363-3p, were upregulated (Figure 2C), and 18 miRNAs were downregulated, including miR-433-3p and miR-496-3p (Figure 2D).

### Quantitative RT-PCR for miRNA

**Figure 3:**
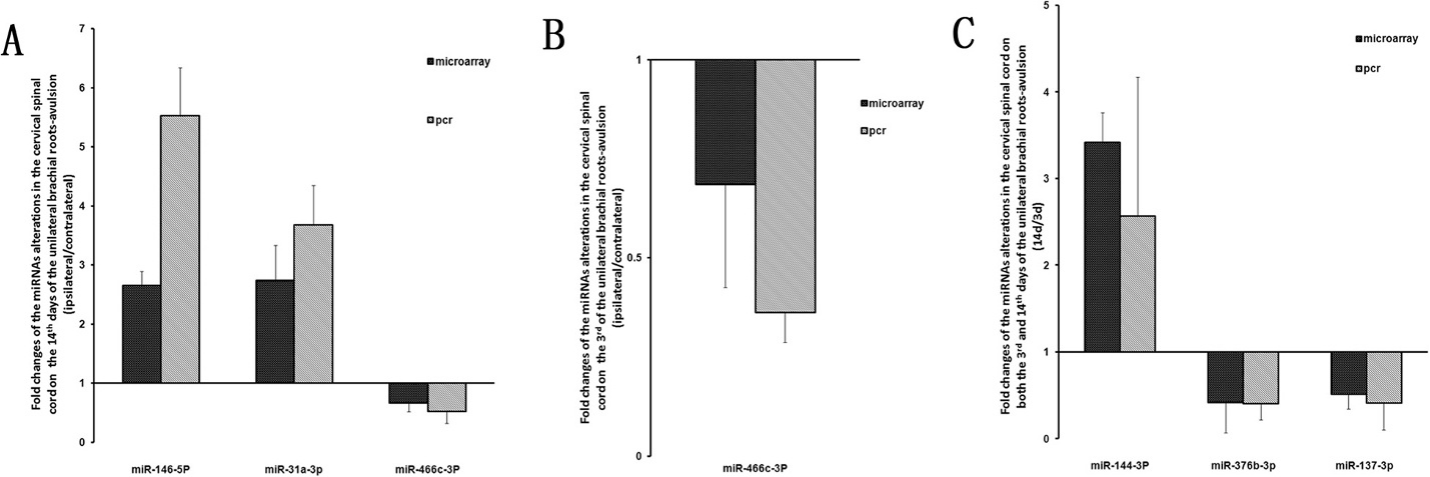
**Temporal expression profiles of selected microRNAs.** The fold changes of the selected microRNAs by quantitative PCR (right columns) compared with the corresponding microarray data (left columns). **(A, B)** The relative expression level on the ipsilateral side relative to the contralateral side **(C)** and the expression on the 3rd day compared to the 14th day on the ipsilateral side. Bars represent the means ± standard error of 3 independent experiments.

To validate the above microarray results, we used qRT-PCR to confirm the expression levels of 6 miRNAs. The qRT-PCR analysis confirmed that miR-146b-5p and miR-31a-3p were significantly upregulated in the ipsilateral spinal cord compared to the contralateral spinal cord on the 14th day after injury (Figure 3A). The expression level of miR-466c-3p was downregulated on both the 3rd and the 14th days after injury (Figure 3B). The expression levels of miR-376b-3p and miR-137-3p were downregulated, but miR-144-3p was upregulated on the 14th day compared to the 3rd day after injury (Figure 3C).

### Bioinformatics analyses of the miRNA targets

According to the results of the miRNAs microarray analysis, the mechanism of the ventral combined with dorsal root avulsion-induced changes in the miRNA expression patterns were further explored by the Gene Ontology project, which was used to evaluate the genes affected by the upregulated and downregulated miRNAs both in the early (the 3rd day) stage and in the intermediate stage (the 14th day, when motoneuron death occurred) after the injury. Based on a cluster analysis of the enriched biological themes, the enrichment score is based on the mean value of the log of the p-values (EASEscore) for the members in that cluster. A cut-off value of 2.0 was used, which corresponds to a p-value <0.01. Thus, a higher enrichment score value indicates a low p-value and therefore indicates that the cluster is significant [14]. The enriched clusters (significantly regulated) related to the following processes were observed on both the 3rd day and 14th day after ventral combined with dorsal root avulsion: ion, amino acid and protein transport; response to organic substances; angiogenesis; cell migration; homeostatic processes; regulation of catalytic activity, phosphorylation and protein kinase cascades; activation of MAPK activity; regulation of cell death and apoptosis; regulation of cell morphogenesis; neuron projection development and axonogenesis; and transmission of nerve impulses and synaptic transmission (Additional file 1: Table S1 and Additional file 2: Table S2). Enriched themes related to the inflammatory response involved on the 3rd day after ventral combined with dorsal root avulsion (Additional file 1: Table S1) and on the 14th day after ventral combined with dorsal root avulsion induced genes related to neurotransmitter transport and the regulation of the I-kappaB kinase/NF-kappaB cascade (Additional file 2: Table S2).

Additionally, we assessed these target genes with KEGG terms. The pathways with p values < 0.05 are listed in Tables 1 and 2. In total, 11 pathways were significantly involved on both the 3rd and 14th days after injury, including the MAPK, calcium and chemokine signaling pathways (Tables 1 and 2). Apoptotic, p53 and T cell receptor signaling pathway responses on the 3rd day after injury (Table 1) were observed, whereas on the 14th day after injury, the activation of the TGF-beta and GnRH signaling pathways was observed (Table 2).

**Figure 4:**
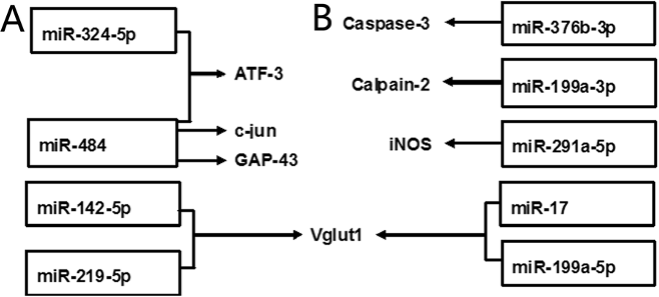
**The potential targets of altered miRNAs following avulsion. (A)** Several genes are potential targets of altered miRNAs on the 3rd day after avulsion. ATF3: activating transcription factor 3; GAP-43: growth associated protein-43. **(B)** Several genes are potential targets of altered miRNAs on the 14th day after ventral combined with dorsal root avulsion. iNOS: inducible nitric oxide synthase.

The predicted target genes of miR-484 and miR-324-3p, which were downregulated on the 14th day, indicate that they potentially targeted activating transcription factor 3 (ATF3), growth associated protein-43 (GAP43) and c-jun (Figure 4A). Caspase-3 was a potential target of the downregulated miR-376b-3p, calpain 2 was a potential target of the upregulated miR-199a-3p, and iNOS was a potential target of downregulated miR-291a-5p (Figure 4B). While miR-142-5p and miR-219-5p were upregulated on the 3rd day after ventral combined with dorsal root avulsion, miR-17 and miR-199a-5p were upregulated on the 14th day after ventral combined with dorsal root avulsion and were predicted to target VGLUT1.

**Table 1:** **Pathways identified by KEGG pathway analysis on the 3rd day after ventral combined with dorsal root avulsion**

Pathway	Count	p-value
Calcium signaling pathway	33	3.11E-04
MAPK signaling pathway	43	4.35E-04
Amyotrophic lateral sclerosis (ALS)	15	8.53E-04
Adipocytokine signaling pathway	16	0.001076
Apoptosis	18	0.001945
PPAR signaling pathway	16	0.002004
Long-term depression	15	0.003117
Renal cell carcinoma	15	0.004136
Long-term potentiation	14	0.008307
Tight junction	22	0.008915
Fc gamma R-mediated phagocytosis	16	0.01576
Chemokine signaling pathway	26	0.015924
Neuroactive ligand-receptor interaction	36	0.018913
Cytokine-cytokine receptor interaction	28	0.027292
Glycerophospholipid metabolism	12	0.02741
Oocyte meiosis	18	0.0284
Ether lipid metabolism	8	0.035229
Gap junction	14	0.036906
p53 signaling pathway	12	0.041325
Natural killer cell mediated cytotoxicity	16	0.044603
Alzheimer's disease	27	0.044866
T cell receptor signaling pathway	17	0.046169
ABC transporters	9	0.049088

**Table 2:** **Pathways identified by KEGG pathway analysis on the 14th day after ventral combined with dorsal root avulsion**

Pathway	Count	p-value
MAPK signaling pathway	42	1.18E-03
Renal cell carcinoma	16	1.76E-03
Glycerophospholipid metabolism	14	4.85E-03
PPAR signaling pathway	15	0.006288011
Calcium signaling pathway	29	0.007027411
Gap junction	16	0.008668647
Long-term potentiation	14	0.009540218
Fc gamma R-mediated phagocytosis	16	0.018173645
Long-term depression	13	0.022920928
Pancreatic cancer	13	0.028323405
GnRH signaling pathway	16	0.031376205
TGF-beta signaling pathway	15	0.03163992
Chemokine signaling pathway	25	0.033529125
Colorectal cancer	14	0.041518293
Fatty acid metabolism	9	0.0419816
Leukocyte transendothelial migration	18	0.044056211

### The expression patterns of altered miRNA-targeted genes in injured spinal cord

**Figure 5:**
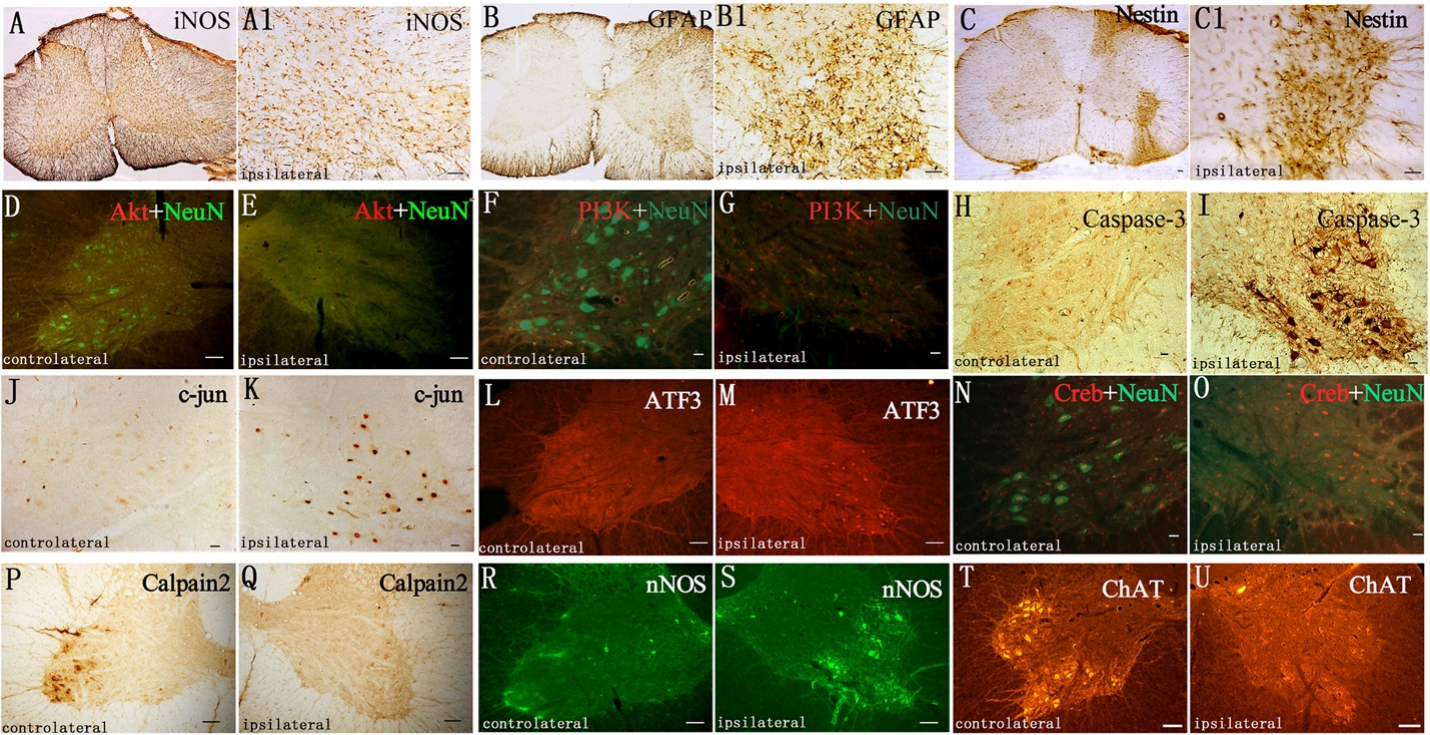
**The expression patterns of altered miRNA target genes in injured spinal cord.** **A**–**C1**: Representative microphotographs of the glial cells on the 14th day after ventral combined with dorsal root avulsion. **A1**, **B1**, **C1** shows the enlarged photographs of **A**, **B**, **C**, respectively. The increased expression of iNOS protein was revealed in injured spinal segments **(A)** and was widely distributed both in the gray and white matter **(A1)**. More obviously, GFAP immunoreactivity was observed in the gray matter of the ipsilateral ventral horns; **(B)** and was activated with larger cell bodies and short processes **(B1)**. The ventral combined with dorsal root avulsion-induced nestin immunoreactivity was concentrated in the Rexed IX layer of the gray matter; **(C)** and showed a spider shape, larger cell bodies, and coarse and short processes **(C1)**. **D**–**I**: Representative microphotographs of the Akt/PKB signaling pathway on the 14th day after ventral combined with dorsal root avulsion. Both Akt **(D, E)** and PI3K **(F, G)** were highly expressed in the glial cells in the bilateral ventral horns. The caspase-3 immunoreactivity was remarkably induced within the cytoplasm of the ipsilateral ventral horn motoneurons **(H, I)** **J**–**O**: Representative microphotographs of c-jun, ATF-3 and CREB. c-jun was highly expressed in the nuclei of the ipsilateral ventral horn motoneurons **(J, K)**. CREB protein was distributed in the nuclei of both motoneurons and glial cells in the bilateral ventral horns **(N, O)**. However, the expression of ATF-3 was only observed inside the motoneurons of the ipsilateral ventral horns **(L, M)**. **P**–**U**: Representative microphotographs of calpain 2, nNOS and ChAT. Ventral combined with dorsal root avulsion resulted in a clear decrease in the expression of calpain-2 in the ipsilateral ventral horn **(P, Q)**. The induction of nNOS was observed only in the ipsilateral ventral horn motoneurons **(R, S)**. Ventral combined with dorsal root avulsion resulted in the disappearance of ChAT in the ipsilateral motoneurons **(T, U)**. Scale bar = 100 μm.

Subsequently, immunohistochemistry and immunofluorescence analyses of target genes predicted by the altered miRNAs were performed after ventral combined with dorsal root avulsion (Figure 5). The activation of glial cells in the spinal cord was revealed by the iNOS, GFAP, and nestin immunoreactivity on the 14th day after ventral combined with dorsal root avulsion (Figure 5A-C1). The increased expression of iNOS protein was observed in injured spinal segments (Figure 5A), especially the ipsilateral half of the cervical spinal cord; most of the iNOS-positive glial cells were distributed widely both in the gray and white matter of the spinal cord (Figure 5A1). More obviously, GFAP immunoreactivity was observed in the gray matter of the ipsilateral ventral horns surrounding the cell bodies of the motoneurons (Figure 5B). The GFAP-positive astrocytes were activated with remarkably larger cell bodies and short processes and were distributed in the peripheral circle of the white matter of the ipsilateral anterior and posterior funiculi (Figure 5B1). The ventral combined with dorsal root avulsion-induced nestin immunoreactivity within the spinal cord was concentrated in Rexed IX layer of the gray matter and in the anterior and posterior funiculi, where the ventral and dorsal roots were localized, respectively (Figure 5C). Similarly, nestin-positive glial cells showed an excessively activated state and were spider-shaped with larger cell bodies and coarse and short processes in the ventral horn containing the lesion and the superficial layer of the dorsal horn (Figure 5C1). The Akt/PKB signaling pathway was found to be involved in the glial activation after ventral combined with dorsal root avulsion. Both Akt (Figure 5D, 5E) and PI3K (Figure 5F, G) were highly expressed in the glial cells in the bilateral ventral horns of the injured spinal segment. The caspase-3 immunoreactivity was absent in the contralateral ventral horn motoneurons (Figure 5H) but was remarkably induced within the cytoplasm of the ipsilateral ventral horn motoneurons (Figure 5I) on the 14th day after unilateral root avulsion. Another member of the activating transcription factors, c-jun, was also highly expressed in the nuclei of the ipsilateral ventral horn motoneurons (Figure 5J-K). The members of the activating transcription factor/cAMP-responsive element binding protein (ATF/CREB) family of transcription factors were induced and were expressed in the nuclei of the cells in the spinal cord (Figure 5L-O). The CREB protein was distributed in the nuclei of both motoneurons and glial cells in the bilateral ventral horns (Figure 5N-O). However, the expression of ATF-3 was only observed within the motoneurons of the ipsilateral ventral horns (Figure 5L-M). Ventral combined with dorsal root avulsion led to the dysfunction of both enzymes and the cytoskeletal reorganization of the injured motoneurons. Ventral combined with dorsal root avulsion resulted in a clear decrease in the expression of calpain-2, the calcium-dependent protease (Figure 5P), in the ipsilateral ventral horn (Figure 5Q). The obvious induction of nNOS was observed only in the ipsilateral ventral horn motoneurons (Figure 5R-S). In the contralateral ventral horn motoneurons, the motoneuron marker (ChAT, Figure 5T) was detected in the cytoplasm of the motoneurons. Ventral combined with dorsal root avulsion resulted in the disappearance of ChAT (Figure 5U) in the ipsilateral motoneurons until the 14th day after the injury.

### The survival rate of motoneurons after ventral combined with dorsal root avulsion

**Figure 6:**
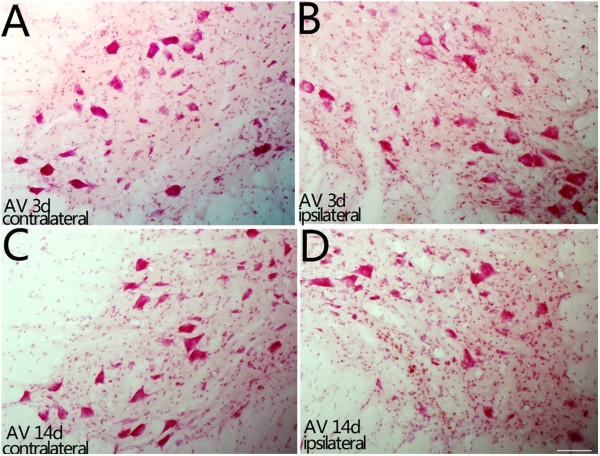
**Effect of ventral combined with dorsal root avulsion on the survival rate of motoneurons in the C7 segment spinal cord.** Representative micrographs of ipsilateral C7 spinal cross-sections showing the survival of motoneurons in the ventral horn on the 3rd day post-injury **(A-B)** and on the 14th day post-injury **(C-D)**. Cross-sections of the spinal cord showing the lateral ventral horn region. Neurons with a large soma and typical Nissl bodies within the dotted line region were counted as surviving motoneurons. The numbers of motoneurons in the contralateral side **(A, C)** and the lesion **(B, D)** sides were counted under a 20x objective lens. Scale bar = 100 μm.

On the 3rd and 14th days after ventral combined with dorsal root avulsion, the survival of the motoneurons in both the ipsilateral and contralateral C7 ventral horns was investigated in neutral red-stained slides. The quantitative results showed that the ipsilateral C7 ventral horn contained 93.2 ± 3.12% as many surviving motoneurons as the contralateral C7 ventral horn on the 3rd day (Figure 6A-B) and 69.08 ± 2.71% on the 14th day(Figure 6C-D) after ventral combined with dorsal root avulsion.

## Discussion

Previous studies have demonstrated that in a spinal root avulsion injury model, the up-regulation of neuronal nitric oxide synthase (nNOS) coincides with the death of spinal motoneurons [15, 16], and the upregulation of c-jun coincides with the regeneration of spinal motoneurons [15, 17]. Furthermore, the peak expression of nNOS induced by ventral combined with dorsal root avulsion in the ipsilateral ventral horn motoneurons occurred from day 14 to day 21 [18, 19], whereas the expression of c-jun activated by ventral combined with dorsal root avulsion peaked between 12 h and 3 d [19]. Given the time course of gene changes and the death of motoneurons, 3 days and 14 days were chosen as the time points for exploring miRNA dysregulation after ventral combined with dorsal root avulsion.

We compared our results to published data from several microarray studies of spinal cord injury. These comparisons revealed that the highly expressed miRNAs from our study were also reported in the study by Timo Brandenburger et al. [20]; they showed that miR-124, the let-7 family and miR-34b-3p belonged to the group of highly expressed miRNAs in the rat spinal cord. These highly expressed miRNAs may be the result of the alteration of different cell populations. This alteration could explain the down-regulation of the neuron-specific miRNA, miR-124 [21]; similarly, astrocyte activation upon injury might be a consequence of the up-regulation of miR-21 [22]. In addition, immune cell infiltration might be related to the down-regulation of miR-181a expression [23].

We also observed good agreement between the present results and the data from Nai-Kui Liu et al. [24] in the miRNA expression pattern. Of the 30 miRNAs they found upregulated in traumatic spinal cord injury, miR-223, miR-214, miR-20b-5p, miR-17, miR-146a, miR-199a-3p, miR-221-3p, miR-146b, and miR-145 were also upregulated in our study, and among the 16 downregulated miRNAs in traumatic spinal cord injury, miR-34a and miR-338 were also downregulated after ventral combined with dorsal root avulsion in our study.

Furthermore, we investigated gene expression on the 3rd day after ventral combined with dorsal root avulsion reported in Yang's study [25], which showed decreased expression of the genes that are known to facilitate neuronal survival and axonal regeneration. Based on our research, miR-484, which was downregulated on the 3rd day after ventral combined with dorsal root avulsion, was shown to modulate axonal regeneration through the target GAP-43 gene.

Brachial plexus avulsion also leads to severe intractable neuropathic pain. When both the dorsal and ventral roots are injured, reproducible pain-related behavior in animal models of radiculopathy can be reproduced [26–29]. We found that miR-21 and miR-221 were upregulated in the ipsilateral ventral horn after ventral combined with dorsal root avulsion. This result is consistent with the study by Bin Yu et al., who showed that miR-21 and miR-221 were upregulated in the dorsal root ganglia after resection of the sciatic nerve in rats [30]. The study by Yuuki Genda et al. also revealed that certain miRNAs, including miR-500, -221 and -21, were related to neuropathic pain in a chronic constriction injury rat model [31].

Furthermore, we also identified the target gene that is related to neuropathic pain mediated by the altered miRNAs after ventral combined with dorsal root avulsion. The predicted target gene of downregulated miR-291a-5p was inducible nitric oxide synthase (iNOS). Subsequently, the expression of iNOS, GFAP and nestin were detected in the spinal cord on the 14th day after ventral combined with dorsal root avulsion. A C5-T1 ventral combined with dorsal root avulsion injury results in the significant activation of microglia and astrocytes in the peripheral circle of the white matter of the ipsilateral anterior and posterior funiculi. Deafferentation clearly influenced the extent of microglial activity in the spinal cord, most likely as a result of partial damage to the spinal cord from ventral root avulsion combined with dorsal root avulsion [29]. Microglial activation also occurred in the contralateral spinal cord; D. J. Chew [29] has attributed this effect to transneuronal collateral mechanisms or ‘reactivity overspill’, which are the most dominant hypotheses [32, 33].

Some studies have reported that the dorsal root lesion alone results in a series of gene changes including not only astroglial and microglial reaction but also the loss of vesicular glutamate transporter 1 (VGLUT1) inputs. Dorsal root avulsion disrupted the primary afferents to the spinal motoneurons, which resulted in glutamatergic input lesions. Llewellyn-Smith IJ et al. showed that the transection reduced the density of VGLUT1-immunoreactive axons in sympathetic subnuclei [34]. The loss of VGLUT1 also occurred following dorsal rhizotomy [35]. In addition, VGLUT1 immunoreactivity in the dorsal horn of the L6-S1 of the spinal cord also decreased following lumbosacral ventral root avulsion [36]. We propose that the dorsal root avulsion-induced loss of VGLUT1 might also contribute to the miRNA alterations in the present study. In the present study, by using the miRWalk database, we determined that the following altered miRNAs target the VGLUT1 gene: miR-142-5p, miR-219-5p, miR-17 and miR-199a-5p. miR-142-5p and miRNA-219-5p, which were upregulated on the 3rd day; and miR-17 and miR-199a-5p, which were upregulated on the 14th day. The upregulation of the above miRNAs might result in the downregulation of VGLUT1 gene expression in the injured spinal cord.

A single miRNA can act on various mRNAs, and an mRNA can also be regulated by multiple miRNAs. Thus, the significance of miRNA dysregulation could be better understood by investigating the complex pathways in which they are involved [37]. The biological analysis of altered miRNAs revealed that the important themes that were affected in both the early and intermediate stages of the injury were related to cell death; synaptic transmission; ion, amino acid and protein transport; regulation of catalytic activity, phosphorylation and the protein kinase cascade.

The data in the study by Marten Risling et al. [14] suggest that the inflammatory response is more prominent 24 h after animals are subjected to avulsion. Our data also suggest that the inflammatory response was more prominent in the early stage of the injury and that the T cell receptor signaling pathway was involved on the 3rd day after ventral combined with dorsal root avulsion. In T lymphocytes, the serine/threonine kinase Akt (also known as protein kinase B) (Akt/PKB) is activated upon T-cell receptor activation or in response to an active form of phosphatidylinositide (PI) 3-kinase [38]. Activated Akt/PKB then suppresses the generation of regulatory T cells [39]. However, immunofluorescence showed that ventral combined with dorsal root avulsion induced little change in the expression of Akt and PI3K in the spinal segment and that both Akt and PI3K were highly expressed in the glial cells in the bilateral ventral horns of the injured spinal segment on the 14th day. Moreover, downstream of PI3K/Akt, caspase 3 was substantially increased in the cytoplasm of the ipsilateral ventral horn motoneurons, consistent with neuronal death [40]. The increase in caspase 3 was consistent with its predicted miRNA, miR-376b-3p, which was downregulated on the 14th day compared to the 3rd day after avulsion.

The pathway most involved on the 14th day after injury was the GnRH signaling pathway. The GnRH receptor activates phospholipase C, which transmits its signal to diacylglycerol (DAG) and inositol 1,4,5-trisphosphate (IP3). Then, DAG activates the intracellular protein kinase C (PKC) pathway, leading to the activation of mitogen-activated protein kinases (MAPKs), and IP3 stimulates the release of intracellular calcium. Both the MAPK and calcium signaling pathways were consistently activated 3 or more days after injury. As members of the MAPK family, ERK1/2 and JNK participate in neuronal survival, regeneration, development and death [41–44]. ERK1/2 may involve the activation of CREB [45], which was distributed in the nuclei of both motoneurons and glial cells in the bilateral ventral horns. However, a member of the ATF/CREB family of transcription factors, ATF3, was only observed in the nuclei of the motoneurons in the ipsilateral ventral horns. The present study found that the downregulated miR-484 and miR-324-3p on the 3rd day after injury potentially targeted ATF3. Moreover, ATF-3 regulates transcription by binding to DNA sites as a homodimer or heterodimer with Jun proteins [46]. Some studies have shown that ATF-3 enhanced c-Jun-mediated neurite formation in PC12 and Neuro-2a neuronal cell lines [47] and in NGF-deprived neonatal superior cervical ganglion neurons [48, 49]. The phosphorylation of the AP-1 protein c-Jun on Ser-63 and Ser-73 by JNK causes increased transcriptional activity [50]. In addition, c-Jun is one of the earliest and most consistent markers of neurons that respond to nerve-fiber transection, and its expression can be related to both degeneration and survival including target re-innervation [42]. In our previous studies, we found altered JNK/c-jun gene expression in avulsion-injured spinal cord [1], and after ventral combined with dorsal root avulsion, c-jun was highly expressed in the nuclei of the ipsilateral ventral horn motoneurons. The predicted target genes further illustrated that the avulsion-induced downregulation of miR-324-3p might be the upstream molecular trigger of the JNK/c-jun pathway because miR-324-3p negatively modulates the c-jun gene.

Another pathway, the Ca^2+^/calmodulin dependent signaling pathway, is involved in regulating synaptic plasticity and dendritic morphology [51] as well as the pathological process of neuronal cell death [52, 53]. Both calpain and nNOS belong to Ca^2+^/calmodulin-dependent enzymes [54]. Calpain has been implicated in neuronal injury, and nNOS is proteolytically cleaved by calpain [55]. Although no miRNA in our study was predicted to target nNOS, we found that the upregulated miR-199a-3p on the 14th day after injury potentially targeted calpain 2, and the immunohistochemistry results showed that ventral combined with dorsal root avulsion resulted in a clear decrease in the expression of calpain 2. In addition, neurotransmitter transport was observed on the 14th day after ventral combined with dorsal root avulsion, and many previous studies have shown that miRNAs may regulate specific neurotransmitter systems [56]. For instance, the overexpression and knockdown of miR-181a in primary neurons revealed the effectiveness of miR-181a in the regulation of the GluR2 subunit of the AMPA receptor [57], a key factor in synaptic plasticity. Dopaminergic receptors can also be regulated by miR-142-3p. Using deletion and site-directed mutagenesis approaches, it was shown that the post-transcriptional regulation of the D1 receptor is specifically mediated by miR-142-3p [58].

In addition, based on comparisons of the ipsilateral ventral horn to the contralateral ventral horn on the 3rd and 14th days after ventral combined with dorsal root avulsion, the number of upregulated miRNAs decreased, whereas the number of downregulated miRNAs remained constant, unlike in some other studies. In contusion SCI models in rats, as the injury response progressed, the number of miRNAs that were downregulated gradually increased, whereas the number of upregulated miRNAs did not change significantly [59]. A similar conclusion was drawn by Strickland et al. [60], who reported that the number of downregulated miRNAs was higher on day 14 than on day 4 post-injury. These observed differences in miRNA expression between studies might be the result of different injury types and severity. Because decreased gene expression was greatest after severe injury [61], extravertebral ventral root avulsion combined with dorsal root avulsion limited the damage outside of the vertebral lamina, but the damage was not as severe as central spinal cord injury. Therefore, the extent of decreased miRNA expression induced by ventral combined with dorsal root avulsion was not substantially altered.

## Conclusions

In the present study, we explored miRNA expression profiles using a brachial ventral combined with dorsal root avulsion injury model and described the pattern of miRNA expression following spinal cord injury. The miRNA expression changes in this study were consistent with previous studies, although discrepancies existed. The present data revealed previously unknown time-specific alterations of a large set of miRNAs in the spinal cord after ventral combined with dorsal root avulsion. The altered miRNA target genes encode components involved in inflammation at the beginning of injury and MAPK and calcium signaling pathway components at the start of motoneuron death. Our results indicate that the alterations in miRNAs may contribute to the mechanism of injured motoneuron degeneration. The manipulation of the altered miRNAs before the start of motoneuron death within 2 weeks in adult rats might be a useful and reliable strategy for inhibiting the subsequent apoptosis and promoting axonal regenerative processes in the treatment of brachial root avulsion.

## Methods

### Surgery

Adult male Sprague–Dawley rats (180–250 g) were obtained from the Laboratory Animal Center of Sun Yat-sen University. All surgical procedures were conducted aseptically in accordance with the Chinese National Health and Medical Research Council (NHMRC) animal ethics guidelines and approved by the Sun Yat-sen University Animal Experimentation Ethics Committee. All rats had free access to water and food. The root avulsion of the right brachial plexus was performed according to the procedures described previously [13, 18]. Briefly, animals were anesthetized by intraperitoneal injections of 10% chloral hydrate (350 mg/kg). In the supine position, the right brachial plexus was exposed, and the right C5-8 and T1 spinal nerve roots were isolated under a surgical microscope [Chenghe microsurgical instruments factory, China]. Extra-vertebral ventral combined with dorsal root avulsion was performed by pulling all of the dorsal and ventral roots of each spinal nerve out with microhemostatic forceps. The avulsed ventral and dorsal roots together with the dorsal root ganglia were cut away from the distal ends of the spinal nerves and confirmed under the microscope. For the sham-operated group, the surgery was performed until the isolation of the right C5-8 and T1 spinal nerve root. Then, the surgical wound was sutured in layers.

### Isolation of RNA from the C5-T1 spinal cord

The avulsion-treated and control rats were sacrificed on the 3rd day or the 14th day after root avulsion. Under the surgical microscope, the cervical spinal cords were exposed, and the ipsilateral half of the spinal cord segments were confirmed with a complete ventral and dorsal root avulsion. Then, the C5 to T1 spinal segments were removed and dissected into ipsilateral and contralateral halves as quickly as possible [3]. Total RNA was isolated using TRIzol (Invitrogen, China) and a miRNeasy mini kit (QIAGEN, China) according to the manufacturer’s instructions.

### MicroRNA array and data analysis

The procedures for the microarray analysis of miRNA expression were described previously [59, 60]. miRNA expression profiling of the avulsion and sham spinal cords were compared by using the miRCURY LNA Array (version 11.0) system (Exiqon, Vedbaek, Denmark). Samples were pooled in equal concentrations to eliminate outliers and were compared to each avulsion and sham sample individually [60]. A total of 12 arrays were labeled with the Exiqon miRCURY Hy3/Hy5 power labeling kit and hybridized on the miRCURY LNA Array (version 11.0) station. Scanning was performed with the Axon GenePix 4000B microarray scanner (Axon Instruments, Foster City, CA, USA). GenePix pro version 6.0 (Axon) was used to read image raw intensity. The intensity of the green signal was calculated after background subtraction, and replicated spots on the same slide were averaged to obtain median intensity. The median normalization method was used to acquire normalized data (foreground minus background divided by median). The median was the 50th percentile of miRNA intensity and was >30 in all samples after background correction. The threshold value for significance used to define upregulation or downregulation of miRNAs was a fold change >1.5. The miRNAs selected for investigation in our study were further filtered based on expression levels and previously published data [62].

### MicroRNA-qPCR assay

The procedures of the microarray analysis of the miRNA expression were described previously [59]. Microarray data were validated using quantitative reverse transcription PCR (qRT-PCR) for miRNAs based on the manufacturer’s protocol (TAKARA, China). The RNA samples were converted to cDNA, and qRT-PCR was performed using the following conditions: 95°C for 30 s and 40 cycles of 95°C for 5 s and 60°C for 30 s using the iCycler iQ5 detection system (Bio-Rad, Hercules, CA, USA). Forward and reverse primers (TAKARA, China) for miR-146b-5p, miR-466c-3p, miR-31a-3p, miR-137-3p, miR-376b-3p and miR-144-3p were used for PCR amplification, and real-time data were normalized using U6 RNA. Relative miRNA expression was determined by calculating the mean difference between the cycle thresholds of the miRNA and the U6 control for each sample [Δcycle threshold (ΔCT)] within each sample group (samples with the same miRNA ID, time, and condition parameters) and expressed as -ΔCT for the relative change in expression. Fold change in miRNA expression was determined by calculating the difference between the mean ΔCTs of avulsion and the sham sample groups at the same time point and spinal cord location (ΔΔCT) and was expressed as fold-change (2^-ΔΔCT^) [60]. The difference in cycle threshold change (-ΔΔCT; between injured subjects at 3 and 14 days post-injury, and the sham controls) was determined using Student’s two-tailed t-test, and the p value was considered significant at 0.05.

### Target prediction

Target prediction of altered miRNAs was performed by using the miRWalk database (http://www.ma.uni-heidelberg.de/apps/zmf/mirwalk/), which is a comprehensive database of miRNAs that contains predicted as well as validated miRNA binding sites and information on all known human, mouse and rat genes [63]. Statistically significant miRNA-mRNA relationships were extracted from the MiRWalk results using two criteria: an independently calculated Poisson p-value <0.05 for multiple binding sites in a predicted gene and identification by at least three of the selected target prediction algorithms [4].

### Immunohistochemistry and immunofluorescence

The target genes of the altered miRNAs were identified in the cervical spinal cord of the ventral combined with dorsal root avulsion-injured rats based on immunohistochemistry (IHC) or immunofluorescence (IF) methods using their antibodies in situ. The primary mouse anti-calpain-2, mouse anti-iNOS, mouse anti-nestin, rabbit anti-Caspase-3, mouse anti-nNOS, and rabbit anti-ATF-3 antibodies were purchased from Santa Cruz Biotechnology (Dallas, Texas, USA); mouse anti-GFAP was from Sigma (St. Louis, MO, USA), and rabbit anti-c-jun, rabbit anti-PI3K, rabbit anti-Akt, and rabbit anti-Erk were purchased from Cell Signaling (Danvers, MA, USA). Goat anti-ChAT and mouse anti-NeuN were purchased from Millipore Cooperation (Billerica, MA, USA). The secondary biotinylated anti-rabbit IgG antibodies were purchased from Boster (Wuhan, China); anti-mouse IgG was purchased from Millipore Cooperation (Billerica, MA, USA), and FITC-conjugated anti-mouse IgG, TRITC-conjugated anti-Rabbit IgG, and TRITC-conjugated anti-goat IgG were purchased from Sigma (St. Louis, MO, USA). The ABC reagents were purchased from Boster (Wuhan, China). The IHC and IF procedures were performed according to our previous publications [1, 2, 19]. Briefly, animals were deeply anesthetized with a lethal dose of chloral hydrate and perfused trans-cardially with normal saline followed by 4% paraformaldehyde in 0.1 M phosphate buffer (PB, pH 7.4). After perfusion, the vertebral column was dissected, and the spinal cord was removed. The C7-C8 spinal segments of each animal were removed, fixed by immersion in 4% paraformaldehyde (4% PFA), and stored in 30% (v/v) sucrose solution in PB overnight. Frozen transverse sections (35 μm) were cut and collected in 0.01 M PBS. Every sixth section was used for iNOS, GFAP, nestin, caspase-3, c-jun and calpain-2 IHC study or ATF-3, nNOS, PI3K, Akt, ChAT and NeuN IF labeling. For IHC staining, sections were washed three times with 0.01 M PBS for 10 min and incubated in 0.3% peroxide in methanol (100%) at room temperature for 15 min to quench endogenous peroxidase activity. After washing in PBS, the sections were incubated in 3% BSA and 0.3% Triton X-100 in 0.01 M PBS at room temperature for 30 min and incubated for 72 h at 4°C with the following primary antibodies: anti-calpain-2 (1:500), anti-iNOS (1:1000), anti-nestin (1:1000), anti-GFAP (1:3000), anti-caspase-3 (1:1000), or anti-c-jun (1:400). After washing in PBS, sections were incubated in the secondary antibodies of anti-rabbit IgG (1:500) or anti-mouse IgG (1:500) at room temperature for 2 h. Then, the sections were rinsed and incubated with ABC reagents (1:500) at room temperature for 45 min. The sections were then washed thoroughly and incubated in 0.05% DAB and 0.01% H_2_O_2_ for 3–5 min until a brown reaction product was observed. For IF labeling, sections were incubated in 3% BSA and 0.3% Triton X-100 in 0.01 M PBS at room temperature for 30 min and incubated with the following primary antibodies for 72 h at 4°C: anti-nNOS (1:2000), anti-PI3K (1:400); anti-Akt (1:400); anti-ChAT (1:500); and anti-NeuN or anti-ATF-3 (1:500). After washing in PBS, the sections were incubated with FITC-conjugated anti-mouse IgG (1:200), TRITC-conjugated anti-Rabbit IgG (1:400), or TRITC-conjugated anti-goat IgG (1:400) at room temperature for 2 h in the dark. After washing in PB, the sections were mounted on glass slides, coverslips were applied with antifade mounting media (50% glycerin in 0.5 M buffer bicarbonate, pH 9.5), and samples were examined via fluorescence microscopy (Zeiss, USA). Control experiments included the omission of the primary or secondary antibodies. Sham-operated animals served as negative controls.

### Bioinformatics of target genes

Lists of predicted target genes for the differentially expressed miRNA families or clusters were uploaded to the Database for Annotation, Visualization, and Integrated Discovery (DAVID, http://david.abcc.ncifcrf.gov) for the functional annotation and detection of enriched functional-related gene groups, and enriched biological themes, particularly GO terms [14], also provided the pathway information.

### Neutral red staining

Neutral red staining of the sections was performed with 1% neutral red (Sigma, St. Louis, MO, USA) for 2 h followed by dehydration in ethanol. The images were captured using a digital camera attached to the microscope (Olympus BX50, Japan) [3].

### Motoneuron counting

Every third section of the C7 spinal segment from each rat was used for neutral red staining. The number of motoneurons was counted on both the intact sides and the injured sides of each C7 spinal segment in neutral red-stained sections by a researcher who was blinded to the animal injury, as described previously [1, 3, 18, 19]. To determine the survival of the motoneurons, we counted the neurons in the Rexed laminae IX in both contralateral and ipsilateral ventral horns because both alpha and gamma motor neurons in these laminae innervate the skeletal muscles. In addition to the motor neurons, there are many interneurons that were stained with neutral red in the Rexed Laminae IX after ventral combined with dorsal root avulsion. The contralateral side was used as an internal control for each section to prevent the influence of the interneurons in cell counting of the motoneurons. According to our previous studies [18, 64], only those neurons with both their nucleolus in the nuclei and their Nissl bodies in the cytoplasm that were stained with neutral red were counted under a 20x objective lens. The numbers of surviving motoneurons in the ipsilateral ventral horn were expressed as percentages of the number of surviving motoneurons in the contralateral ventral horn of the same section in each rat [15, 18], with three rats in each subgroup in the present study.

### Statistical analysis

All data reported represent at least 3 independent experiments for n = 3 samples in each experiment. Data were expressed as the means ± SD. Statistical analysis was performed with Student’s two-tailed t-test (SPSS 16, SPSS Inc., Chicago, IL, USA). P < 0.05 was considered significant.

## Electronic supplementary material

Additional file 1: Table S1: The enriched clusters in avulsion vs. control on the 3rd day post-injury; cluster analysis of regulated genes, employing the Database for Annotation, Visualization, and Integrated Discovery (DAVID). It was possible to group responding genes into functionally related gene groups (GO – Gene Ontology search terms) and clusters of enriched biological themes. The enrichment score is based on the mean value of the - log of the p-values (EASEscore) for the members in that cluster. A cut-off value of 2.0 was used, which corresponds to a p-value <0.01. (XLSX 17 KB)

Additional file 2: Table S2: The enriched clusters in avulsion vs. control on the 14th day post-injury. (XLSX 17 KB)

## Abbreviations

ATF3: Activating transcription factor 3
CREB: cAMP-response-element-binding protein
DAG: Diacylglycerol
GAP-43: Growth associated protein-43
iNOS: Inducible nitric oxide synthase
IP3: Inositol 1,4,5-trisphosphate
MAPK: Mitogen-activated protein kinases
miRNA: microRNA
nNOS: Neuronal nitric oxide synthase
PI3K: Phosphatidylinositide (PI) 3-kinase
PKC: Protein kinase C
VGLUT1: Vesicular glutamate transporter 1.

## References

[1] Wang LL, Zhao XC, Yan LF, Wang YQ, Cheng X, Fu R, Zhou LH (2011). C-jun phosphorylation contributes to down regulation of neuronal nitric oxide synthase protein and motoneurons death in injured spinal cords following root-avulsion of the brachial plexus. Neuroscience.

[2] Zhao XC, Wang LL, Wang YQ, Song FH, Li YQ, Fu R, Zheng WH, Wu W, Zhou LH (2012). Activation of phospholipase-Cgamma and protein kinase C signal pathways helps the survival of spinal motoneurons injured by root avulsion. J Neurochem.

[3] Cheng X, Fu R, Gao M, Liu S, Li YQ, Song FH, Bruce IC, Zhou LH, Wu W (2013). Intrathecal application of short interfering RNA knocks down c-jun expression and augments spinal motoneuron death after root avulsion in adult rats. Neuroscience.

[4] Juhila J, Sipila T, Icay K, Nicorici D, Ellonen P, Kallio A, Korpelainen E, Greco D, Hovatta I (2011). MicroRNA expression profiling reveals miRNA families regulating specific biological pathways in mouse frontal cortex and hippocampus. PLoS One.

[5] Wang J, Yan L, Zhao X, Wu W, Zhou LH (2010). The diversity of nNOS gene expression in avulsion-injured spinal motoneurons among laboratory rodents. Nitric Oxide.

[6] Du T, Zamore PD (2005). microPrimer: the biogenesis and function of microRNA. Development.

[7] Carthew RW, Sontheimer EJ (2009). Origins and Mechanisms of miRNAs and siRNAs. Cell.

[8] Bhalala OG, Srikanth M, Kessler JA (2013). The emerging roles of microRNAs in CNS injuries. Nat Rev Neurol.

[9] Lee CT, Risom T, Strauss WM (2007). Evolutionary conservation of microRNA regulatory circuits: an examination of microRNA gene complexity and conserved microRNA-target interactions through metazoan phylogeny. DNA Cell Biol.

[10] Haramati S, Chapnik E, Sztainberg Y, Eilam R, Zwang R, Gershoni N, McGlinn E, Heiser PW, Wills AM, Wirguin I, Rubin LL, Misawa H, Tabin CJ, Brown R, Chen A, Hornstein E (2010). miRNA malfunction causes spinal motor neuron disease. Proc Natl Acad Sci U S A.

[11] Nelson PT, Wang WX, Rajeev BW (2008). MicroRNAs (miRNAs) in neurodegenerative diseases. Brain Pathol.

[12] Campos-Melo D, Droppelmann CA, He Z, Volkening K, Strong MJ (2013). Altered microRNA expression profile in Amyotrophic Lateral Sclerosis: a role in the regulation of NFL mRNA levels. Mol Brain.

[13] Zhou LH, Wu W (2006). Survival of injured spinal motoneurons in adult rat upon treatment with glial cell line-derived neurotrophic factor at 2 weeks but not at 4 weeks after root avulsion. J Neurotrauma.

[14] Risling M, Ochsman T, Carlstedt T, Linda H, Plantman S, Rostami E, Angeria M, Skold MK (2011). On acute gene expression changes after ventral root replantation. Front Neurol.

[15] Wu W, Li Y, Schinco FP (1994). Expression of c-jun and neuronal nitric oxide synthase in rat spinal motoneurons following axonal injury. Neurosci Lett.

[16] Wu W, Li L, Yick LW, Chai H, Xie Y, Yang Y, Prevette DM, Oppenheim RW (2003). GDNF and BDNF alter the expression of neuronal NOS, c-Jun, and p75 and prevent motoneuron death following spinal root avulsion in adult rats. J Neurotrauma.

[17] Wu W (1996). Potential roles of gene expression change in adult rat spinal motoneurons following axonal injury: a comparison among c-jun, off-affinity nerve growth factor receptor (LNGFR), and nitric oxide synthase (NOS). Exp Neurol.

[18] Wu W (1993). Expression of nitric-oxide synthase (NOS) in injured CNS neurons as shown by NADPH diaphorase histochemistry. Exp Neurol.

[19] Zhou LH, Han S, Xie YY, Wang LL, Yao ZB (2008). Differences in c-jun and nNOS expression levels in motoneurons following different kinds of axonal injury in adult rats. Brain Cell Biol.

[20] Brandenburger T, Castoldi M, Brendel M, Grievink H, Schlosser L, Werdehausen R, Bauer I, Hermanns H (2012). Expression of spinal cord microRNAs in a rat model of chronic neuropathic pain. Neurosci Lett.

[21] Makeyev EV, Zhang J, Carrasco MA, Maniatis T (2007). The MicroRNA miR-124 promotes neuronal differentiation by triggering brain-specific alternative pre-mRNA splicing. Mol Cell.

[22] Bhalala OG, Pan L, Sahni V, McGuire TL, Gruner K, Tourtellotte WG, Kessler JA (2012). microRNA-21 regulates astrocytic response following spinal cord injury. J Neurosci.

[23] Xie W, Li M, Xu N, Lv Q, Huang N, He J, Zhang Y (2013). MiR-181a regulates inflammation responses in monocytes and macrophages. PLoS One.

[24] Liu NK, Wang XF, Lu QB, Xu XM (2009). Altered microRNA expression following traumatic spinal cord injury. Exp Neurol.

[25] Yang Y, Xie Y, Chai H, Fan M, Liu S, Liu H, Bruce I, Wu W (2006). Microarray analysis of gene expression patterns in adult spinal motoneurons after different types of axonal injuries. Brain Res.

[26] Hunt JL, Winkelstein BA, Rutkowski MD, Weinstein JN, DeLeo JA (2001). Repeated injury to the lumbar nerve roots produces enhanced mechanical allodynia and persistent spinal neuroinflammation. Spine (Phila Pa 1976).

[27] Winkelstein BA, Rutkowski MD, Sweitzer SM, Pahl JL, DeLeo JA (2001). Nerve injury proximal or distal to the DRG induces similar spinal glial activation and selective cytokine expression but differential behavioral responses to pharmacologic treatment. J Comp Neurol.

[28] Winkelstein BA, DeLeo JA (2002). Nerve root injury severity differentially modulates spinal glial activation in a rat lumbar radiculopathy model: considerations for persistent pain. Brain Res.

[29] Chew DJ, Carlstedt T, Shortland PJ (2011). A comparative histological analysis of two models of nerve root avulsion injury in the adult rat. Neuropathol Appl Neurobiol.

[30] Yu B, Zhou S, Qian T, Wang Y, Ding F, Gu X (2011). Altered microRNA expression following sciatic nerve resection in dorsal root ganglia of rats. Acta Biochim Biophys Sin (Shanghai).

[31] Genda Y, Arai M, Ishikawa M, Tanaka S, Okabe T, Sakamoto A (2013). microRNA changes in the dorsal horn of the spinal cord of rats with chronic constriction injury: A TaqMan(R) Low Density Array study. Int J Mol Med.

[32] Donaldson LF (1999). Unilateral arthritis: contralateral effects. Trends Neurosci.

[33] Koltzenburg M, Wall PD, McMahon SB (1999). Does the right side know what the left is doing?. Trends Neurosci.

[34] Llewellyn-Smith IJ, Martin CL, Fenwick NM, Dicarlo SE, Lujan HL, Schreihofer AM (2007). VGLUT1 and VGLUT2 innervation in autonomic regions of intact and transected rat spinal cord. J Comp Neurol.

[35] Alvarez FJ, Villalba RM, Zerda R, Schneider SP (2004). Vesicular glutamate transporters in the spinal cord, with special reference to sensory primary afferent synapses. J Comp Neurol.

[36] Wu L, Wu J, Chang HYH, Havton LA (2012). Selective plasticity of primary afferent innervation to the dorsal horn and autonomic nuclei following lumbosacral ventral root avulsion and reimplantation in long term studies. Exp Neurol.

[37] Dharap A, Vemuganti R (2010). Ischemic pre-conditioning alters cerebral microRNAs that are upstream to neuroprotective signaling pathways. J Neurochem.

[38] Genot EM, Arrieumerlou C, Ku G, Burgering BM, Weiss A, Kramer IM (2000). The T-cell receptor regulates Akt (protein kinase B) via a pathway involving Rac1 and phosphatidylinositide 3-kinase. Mol Cell Biol.

[39] So T, Croft M (2013). Regulation of PI-3-Kinase and Akt Signaling in T Lymphocytes and Other Cells by TNFR Family Molecules. Front Immunol.

[40] Xu YQ, Long L, Yan JQ, Wei L, Pan MQ, Gao HM, Zhou P, Liu M, Zhu CS, Tang BS, Wang Q (2013). Simvastatin induces neuroprotection in 6-OHDA-lesioned PC12 via the PI3K/AKT/caspase 3 pathway and anti-inflammatory responses. CNS Neurosci Ther.

[41] Yuan J, Yankner BA (2000). Apoptosis in the nervous system. Nature.

[42] Herdegen T, Skene P, Bahr M (1997). The c-Jun transcription factor–bipotential mediator of neuronal death, survival and regeneration. Trends Neurosci.

[43] Mielke K, Herdegen T (2000). JNK and p38 stresskinases–degenerative effectors of signal-transduction-cascades in the nervous system. Prog Neurobiol.

[44] Castagne V, Gautschi M, Lefevre K, Posada A, Clarke PG (1999). Relationships between neuronal death and the cellular redox status. Focus on the developing nervous system. Prog Neurobiol.

[45] Bonni A, Brunet A, West AE, Datta SR, Takasu MA, Greenberg ME (1999). Cell survival promoted by the Ras-MAPK signaling pathway by transcription-dependent and -independent mechanisms. Science.

[46] Tsujino H, Kondo E, Fukuoka T, Dai Y, Tokunaga A, Miki K, Yonenobu K, Ochi T, Noguchi K (2000). Activating transcription factor 3 (ATF3) induction by axotomy in sensory and motoneurons: A novel neuronal marker of nerve injury. Mol Cell Neurosci.

[47] Pearson AG, Gray CW, Pearson JF, Greenwood JM, During MJ, Dragunow M (2003). ATF3 enhances c-Jun-mediated neurite sprouting. Brain Res Mol Brain Res.

[48] Nakagomi S, Suzuki Y, Namikawa K, Kiryu-Seo S, Kiyama H (2003). Expression of the activating transcription factor 3 prevents c-Jun N-terminal kinase-induced neuronal death by promoting heat shock protein 27 expression and Akt activation. J Neurosci.

[49] Seijffers R, Allchorne AJ, Woolf CJ (2006). The transcription factor ATF-3 promotes neurite outgrowth. Mol Cell Neurosci.

[50] Schroeter H, Spencer JP, Rice-Evans C, Williams RJ (2001). Flavonoids protect neurons from oxidized low-density-lipoprotein-induced apoptosis involving c-Jun N-terminal kinase (JNK), c-Jun and caspase-3. Biochem J.

[51] Fukunaga K, Miyamoto E (2000). A working model of CaM kinase II activity in hippocampal long-term potentiation and memory. Neurosci Res.

[52] Han F, Shirasaki Y, Fukunaga K (2006). Microsphere embolism-induced endothelial nitric oxide synthase expression mediates disruption of the blood–brain barrier in rat brain. J Neurochem.

[53] Shioda N, Han F, Moriguchi S, Fukunaga K (2007). Constitutively active calcineurin mediates delayed neuronal death through Fas-ligand expression via activation of NFAT and FKHR transcriptional activities in mouse brain ischemia. J Neurochem.

[54] Zhang GS, Ye WF, Tao RR, Lu YM, Shen GF, Fukunaga K, Huang JY, Ji YL, Han F (2012). Expression profiling of Ca2+/calmodulin-dependent signaling molecules in the rat dorsal and ventral hippocampus after acute lead exposure. Exp Toxicol Pathol.

[55] Hajimohammadreza I, Raser KJ, Nath R, Nadimpalli R, Scott M, Wang KK (1997). Neuronal nitric oxide synthase and calmodulin-dependent protein kinase IIalpha undergo neurotoxin-induced proteolysis. J Neurochem.

[56] Higa GS, de Sousa E, Walter LT, Kinjo ER, Resende RR, Kihara AH (2014). MicroRNAs in Neuronal Communication. Mol Neurobiol.

[57] Saba R, Storchel PH, Aksoy-Aksel A, Kepura F, Lippi G, Plant TD, Schratt GM (2012). Dopamine-regulated microRNA MiR-181a controls GluA2 surface expression in hippocampal neurons. Mol Cell Biol.

[58] Tobon KE, Chang D, Kuzhikandathil EV (2012). MicroRNA 142-3p Mediates Post-Transcriptional Regulation of D1 Dopamine Receptor Expression. Plos One.

[59] Yunta M, Nieto-Diaz M, Esteban FJ, Caballero-Lopez M, Navarro-Ruiz R, Reigada D, Pita-Thomas DW, del Aguila A, Munoz-Galdeano T, Maza RM (2012). MicroRNA dysregulation in the spinal cord following traumatic injury. PLoS One.

[60] Strickland ER, Hook MA, Balaraman S, Huie JR, Grau JW, Miranda RC (2011). MicroRNA dysregulation following spinal cord contusion: implications for neural plasticity and repair. Neuroscience.

[61] De Biase A, Knoblach SM, Di Giovanni S, Fan C, Molon A, Hoffman EP, Faden AI (2005). Gene expression profiling of experimental traumatic spinal cord injury as a function of distance from impact site and injury severity. Physiol Genomics.

[62] Li S, Zhu J, Zhang W, Chen Y, Zhang K, Popescu LM, Ma X, Lau WB, Rong R, Yu X, Wang B, Li Y, Xiao C, Zhang M, Wang S, Yu L, Chen AF, Yang X, Cai J (2011). Signature microRNA expression profile of essential hypertension and its novel link to human cytomegalovirus infection. Circulation.

[63] Dweep H, Sticht C, Pandey P, Gretz N (2011). miRWalk–database: prediction of possible miRNA binding sites by "walking" the genes of three genomes. J Biomed Inform.

[64] Wu W, Li L (1993). Inhibition of nitric oxide synthase reduces motoneuron death due to spinal root avulsion. Neurosci Lett.

